# Rheological, Structural, and Biological Trade-Offs in Bioink Design for 3D Bioprinting

**DOI:** 10.3390/gels11080659

**Published:** 2025-08-19

**Authors:** Jeevithan Elango, Camilo Zamora-Ledezma

**Affiliations:** 1 Department of Biomaterials Engineering, Faculty of Health Sciences, UCAM-Universidad Católica San Antonio de Murcia, Campus de los Jerónimos 135, 30107 Murcia, Spain; 2 Center of Molecular Medicine and Diagnostics (COMManD), Department of Biochemistry, Saveetha Dental College and Hospitals, Saveetha Institute of Medical and Technical Sciences, Saveetha University, Chennai 600077, India; 3 Bioengineering & Regenerative Medicine Research Group (Bio-ReM), Escuela de Ingeniería, Arquitectura y Diseño (EIAD), Universidad Alfonso X el Sabio (UAX), Avenida de la Universidad 1, Villanueva de la Cañada, 28691 Madrid, Spain

**Keywords:** mesenchymal stem cells, cell-laden bioinks, rheology, biological functionality, 3D bioprinting hydrogels, tissue engineering, viscoelasticity

## Abstract

Bioinks represent the core of 3D bioprinting, as they are the carrier responsible for enabling the fabrication of anatomically precise, cell-laden constructs that replicate native tissue architecture. Indeed, their role goes beyond structural support, as they must also sustain cellular viability, proliferation, and differentiation functions, which are critical for applications in the field of regenerative medicine and personalized therapies. However, at present, a persistent challenge lies in reconciling the conflicting demands of rheological properties, which are essential for printability and biological functionality. This trade-off limits the clinical translation of bioprinted tissues, particularly for vascularized or mechanically dynamic organs. Despite huge progress during the last decade, challenges persist in standardizing bioink characterization, scaling production, and ensuring long-term biomimetic performance. Based on these challenges, this review explores the inherent trade-off faced by bioink research optimizing rheology to ensure printability, shape fidelity, and structural integrity, while simultaneously maintaining high cell viability, proliferation, and tissue maturation offering insights into designing next-generation bioinks for functional tissue engineering.

## 1. Introduction

3D bioprinting has emerged as a groundbreaking technology in tissue engineering and regenerative medicine, offering unparalleled potential for fabricating complex, three-dimensional tissue constructs [1,2,3]. Unlike traditional tissue engineering methods, which often rely on manual assembly and scaffolding techniques, 3D bioprinting enables the precise deposition of biomaterials and living cells in a layer-by-layer fashion, mimicking the intricate structural and functional characteristics of native tissues and organs [4,5]. This technological advancement holds promise for addressing critical medical challenges, such as organ shortages, tissue damage repair, and disease modeling, by creating biomimetic structures tailored to patient-specific needs.

At the core of this innovative approach lies bioinks, a specialized material formulation designed to encapsulate and deliver cells, growth factors, and bioactive molecules to a designated location with spatial control [6,7]. The composition and properties of bioinks are of paramount importance, as they dictate essential factors such as printability, structural fidelity, mechanical stability, and cell viability. A well-formulated bioinks must exhibit optimal rheological behavior, ensuring it can be extruded smoothly during the printing process while maintaining its structural integrity post-printing. Additionally, bioinks should support cell adhesion, proliferation, and differentiation, ensuring that the printed tissue can undergo proper maturation and functional integration with surrounding biological environments.

Despite the tremendous potential of 3D bioprinting, the realization of fully functional, bioprinted tissues and organs remains contingent upon the development of advanced bioinks. Without appropriate bioink formulations, challenges such as poor mechanical stability, insufficient biological interactions, and compromised cell viability hinder the successful translation of bioprinted constructs into clinically relevant applications. Therefore, intensive research efforts are focused on optimizing bioink properties to strike a balance between mechanical robustness and biological functionality, ensuring that engineered tissues exhibit the necessary characteristics for long-term viability and integration.

The significance of bioink development extends beyond regenerative medicine, encompassing diverse applications such as drug screening, disease modeling, personalized tissue implants, and organ replacement [8,9,10]. By providing platforms that mimic physiological conditions, bioprinted tissues enable preclinical drug testing and toxicity assessments, reducing reliance on animal models and enhancing predictive accuracy for human responses. Furthermore, patient-specific tissue constructs hold great promise for precision medicine, enabling tailored treatments based on an individual’s biological profile [11,12,13]. With continuous advancements in biomaterial science, bioengineering, and bioprinting techniques, the evolution of bioinks will remain a cornerstone in the pursuit of next-generation biomedical solutions.

Our recent research claimed that the rheological properties of a bioink play a fundamental role in determining its behavior during the bioprinting process, directly influencing printability, structural integrity, and the overall success of tissue fabrication [14]. Several key rheological parameters—such as viscosity, shear-thinning behavior, and gelation kinetics—must be carefully optimized to ensure the bioink can be precisely extruded, maintain its shape post-deposition, and provide a stable matrix for embedded cells. The viscosity of a bioink defines the ease with which it can flow through the printing nozzle; a high-viscosity bioink may hinder extrusion, requiring excessive force that could compromise cell viability, while a low-viscosity bioink may lead to excessive spreading, resulting in poor print resolution and structural instability [15].

**Figure 1:**
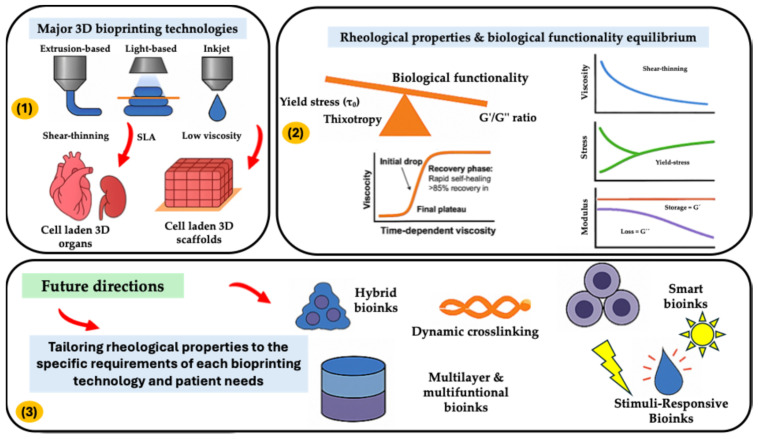
Overall concept of Rheological Properties and Future Directions in Bioprinting Technologies.

A desirable feature in bioink formulation is shear-thinning behavior, wherein viscosity decreases under shear stress, allowing smooth extrusion during printing while recovering its consistency post-deposition (Figure 1). This property is crucial for preventing clogging, ensuring continuous and uniform filament formation, and facilitating high-precision construct fabrication. Additionally, the bioinks must possess gelation kinetics that enables rapid solidification or crosslinking upon printing, stabilizing the printed construct and preventing deformation or collapse before tissue maturation [16,17]. Depending on the specific application, crosslinking may be achieved through chemical, thermal, ionic, or enzymatic processes, each of which influences the bioink’s mechanical strength and cellular environment. However, optimizing the rheological properties alone is insufficient; biological functionality must also be considered to ensure cell viability, adhesion, proliferation, differentiation, and extracellular matrix deposition within the bioprinted structure. The choice of biomaterials used in bioink formulation significantly impacts both rheological and biological performance. Natural polymers such as collagen, alginate, gelatin, and hyaluronic acid provide an environment conducive to cell attachment and tissue remodeling, while synthetic polymers such as polyethylene glycol (PEG) and polycaprolactone (PCL) offer tunable mechanical properties suitable for structural reinforcement [18,19,20,21]. Often, hybrid bioinks combining natural and synthetic components are developed to achieve an optimal balance between biocompatibility and mechanical stability. Moreover, the incorporation of bioactive agents, such as growth factors, cytokines, cell adhesion molecules, and signaling peptides, further enhances the biological functionality of the bioinks, promoting tissue-specific responses and facilitating cell differentiation into functional tissue architectures [22,23,24]. By carefully engineering the interplay between rheological control and biological compatibility, researchers aim to develop next-generation bioinks capable of recapitulating native tissue environments, enabling advancements in tissue engineering, regenerative medicine, and personalized organ fabrication.

A significant challenge in bioink development lies in the inherent trade-off between rheological properties and biological functionality. Optimizing one feature often compromises the other. For instance, increasing the concentration of a polymer to enhance the bioink’s viscosity and mechanical strength may negatively impact cell viability or nutrient diffusion. Similarly, incorporating certain bioactive molecules may alter the bioink’s rheological behavior, making it difficult to print.

This trade-off necessitates a careful and iterative approach to bioink design, where the specific requirements of the target tissue and application are considered. Researchers are exploring various strategies to overcome this challenge, including the use of composite bioinks, which combine multiple materials to achieve a synergistic effect, and the development of stimuli-responsive bioinks, which can be tuned to change their properties in response to external cues.

In this review, we deliberately confine the discussion to hydrogel-based bioinks utilized in extrusion-based 3D bioprinting, as these materials offer optimal compatibility with live cell encapsulation and tissue engineering requirements. Our focus is centered on the rheological, structural, and biological parameters directly influencing print fidelity and cellular function, including crosslinking strategies, viscosity control, and matrix-cell interactions. Technologies such as laser-assisted, inkjet, and scaffold-free bioprinting modalities are excluded, as are formulations intended primarily for mechanical reinforcement without bioactivity. Hardware configurations, bioreactor systems, and long-term in vivo integration methods are referenced only when they intersect with bioink design decisions. Similarly, commercialization, regulatory pathways, and whole-organ printing approaches are considered beyond the scope, ensuring this review maintains a clear emphasis on material-centric trade-offs impacting soft tissue construct viability and maturation. Hence, the major scope of this review focuses specifically on the interdependent relationship between rheological characteristics, structural fidelity, and biological viability in bioink formulations used in extrusion-based, light-based and inkjet-based 3D bioprinting. By narrowing its lens to materials and strategies designed for soft tissue engineering applications, this article critically evaluates recent advances aimed at optimizing printability without compromising cellular health or functional integration.

Understanding and addressing this trade-off is essential for realizing the full potential of 3D bioprinting in tissue engineering and regenerative medicine. This review will explore the critical role of bioink in 3D bioprinting for biomedical applications, examining the interplay between rheological properties and biological functionality, as well as the trade-offs that influence tissue engineering outcomes. By highlighting current advancements and challenges, this review aims to provide significant insights for future research on developing optimized bioinks that enhance structural integrity, cell viability, and functional tissue maturation.

## 2. Fundamentals of Bioinks Rheology

Understanding the rheological behavior of bioinks is essential for optimizing their performance in extrusion-based 3D bioprinting applications), where material flow properties directly influence printability, structural fidelity, and biological functionality [25,26,27]. Thus, in this section key definitions such as viscosity, shear-thinning, yield stress, viscoelasticity (G′, G″), and thixotropy are briefly explained to provide a clear understanding of the importance of these physical phenomena in the fabrication of tailored bioinks for biomedical applications [14,28,29].

### 2.1. Viscosity

It represents the fundamental measure of a material’s resistance to flow under applied shear stress, quantifying the internal friction between fluid layers during deformation. In bioinks formulation, viscosity serves as a critical parameter that governs both printability and post-printing shape retention [30]. For instance, Newtonian fluids maintain constant viscosity regardless of shear rate, in contrast, non-Newtonian bioinks exhibit viscosity variations that depend on applied shear conditions. Thus, optimizing viscosity in bioinks systems requires carefully balancing processability through printing nozzles with the preservation of structural integrity after extrusion. Notably, it has been reported that excessive viscosity can impede material flow and increase extrusion pressures that may damage encapsulated cells. On the contrary, insufficient viscosity compromises the shape fidelity and structural stability of printed constructs [31].

### 2.2. Shear-Thinning Behavior or Pseudoplastic Behavior

It refers to the phenomenon where material’s viscosity decreases with increasing shear rate, thereby enhancing its flow characteristics during extrusion [30,32]. This behavior is particularly advantageous in bioink applications, as it facilitates smooth material flow through printing nozzles under high shear conditions, while maintaining structural integrity at low shear rates following deposition. The mechanism behind this behavior involves structural reorganization of the polymeric networks, where chain entanglements and molecular interactions are disrupted under shear stress, reducing flow resistance. In the literature, it is very common to employ mathematical modeling of shear-thinning behavior by means of power law relationships, where the flow behavior index quantifies the degree of non-Newtonian character. Also, several mathematical models have been successfully used to understand the mechanism in more complex systems. In the context of bioinks, it has been reported that such materials exhibiting pronounced shear-thinning properties demonstrate enhanced printability while preserving cell viability through reduced mechanical stress during processing [32].

### 2.3. Yield Stress

It defines the minimum stress threshold required to initiate material flow, representing the transition point between solid-like and liquid-like behavior. In bioink systems, yield stress determines the material’s ability to maintain structural integrity under gravitational forces and support overhanging features during layer-by-layer construction. Typically, materials exhibiting yield stress behavior remain dimensionally stable until the applied stress exceeds the critical threshold, after which they flow as viscous liquids [30]. In this context, the Herschel-Bulkley model effectively describes yield stress fluids that combine both yield point and shear-thinning characteristics, providing an accurate representation of complex bioinks rheology [30]. For bioink applications, an optimal yield stress value must balance structural support requirements with processability constraints but ensure adequate shape retention without compromising extrudability.

### 2.4. Viscoelasticity (G′/G″)

Viscoelastic properties characterize materials that simultaneously exhibit both viscous and elastic responses to applied deformation, representing the dual nature of bioinks behavior under dynamic conditions. Specifically, the storage modulus (G′) quantifies the elastic component, indicating the material’s ability to store deformation energy and recover its original shape upon stress removal. Conversely, the loss modulus (G′) represents the viscous component, measuring energy dissipation through irreversible deformation and flow. The relative magnitudes of these moduli determine whether a material behaves predominantly as an elastic solid (G′ > G″) or viscous liquid (G″ > G′) [30,33]. A key parameter within the viscoelastic properties of bioink formulation is the loss tangent (tan δ = G″/G′) which provides a dimensionless measure of the material’s damping characteristics and phase behavior. Further, the frequency-dependent viscoelastic properties enable bioinks to exhibit different mechanical responses across various timescales, optimizing both processing behavior and post-printing performance.

### 2.5. Thixotropy

It describes the time-dependent reduction in viscosity under constant shear stress, followed by gradual structural recovery during rest periods. This reversible phenomenon results from the breakdown and reformation of intermolecular networks within the bioink matrix, enabling controlled flow behavior during processing. Thus, thixotropic materials exhibit characteristic hysteresis loops in their flow curves, where the area enclosed by the loop quantifies the degree of structural breakdown. Furthermore, contrary to instantaneous shear-thinning responses, thixotropic behavior involves time-dependent structural changes that can be tailored for specific bioprinting applications. Indeed, the recovery kinetics of thixotropic bioinks must be optimized to allow sufficient flow during extrusion but, at the same time enable rapid structural recovery to maintain printed geometry. This temporal aspect of rheological behavior is particularly valuable for controlling material properties throughout the printing process and subsequent maturation phases [14,28,29]. In Table 1, the main rheological parameters, their impact on bioink formulations, and the ideal rheological ranges for bioinks are summarized. Further, Table 2 shows the key rheological properties governing bioink performance.

### 2.6. Rheological Characterization Methods in Bioink Assessment

The rheological characterization of bioinks requires both fundamental rheometric techniques and application-specific printability assessments to fully evaluate material performance in extrusion-based bioprinting systems [25,32]. First, rotational rheometry serves as the foundation for understanding flow behavior, employing steady-state shear rate sweeps to determine viscosity profiles across physiologically relevant shear conditions typically ranging from 0.1 to 1000 s^−1^. These measurements enable quantification of shear-thinning behavior through power-law fitting, where the consistency index (K) correlates directly with extrusion pressure requirements, and the flow behavior index (n) indicates the degree of pseudoplastic character essential for smooth material flow through printing nozzles. Also, oscillatory rheometry provides complementary viscoelastic characterization through small amplitude oscillatory shear (SAOS) testing, where amplitude sweeps determine the linear viscoelastic region and critical strain values that define material stability under deformation. This is commonly accompanied by frequency sweeps within this linear range which would reveal the relative dominance of elastic (G′) versus viscous (G″) moduli, with the crossover point indicating the transition between solid-like and liquid-like behavior that governs post-extrusion shape retention. In bioink formulations, the printability assessment extends beyond the abovementioned routes, and thus, testing protocols that directly evaluate bioink performance under realistic printing conditions and geometric constraints are in more demand nowadays. The filament collapse test offers a quantitative method for evaluating shape fidelity by extruding suspended filaments across incrementally increasing gap distances. Collapse coefficients are determined as the percentage difference between the theoretical and actual coverage areas. Also, complementary filament fusion testing evaluates lateral spreading and resolution maintenance by printing parallel lines at decreasing spacing intervals, providing a direct measurement of minimum achievable feature sizes and pore structure preservation. Furthermore, these geometric assessments are often combined with automated image analysis methodologies that achieve greater than 95% accuracy in quantifying dimensional parameters including line width, height, aspect ratios, and turn accuracy through differential geometry algorithms. Additional printability metrics include extrudability measurements that quantify pressure requirements for achieving target flow rates, typically establishing minimum flow thresholds for reasonable print times but maintaining maximum pressure limits to preserve cell viability [30].

### 2.7. Rheological Influence on Bioprinting Performance Parameters

The rheological properties of bioink exert interconnected influences on extrusion dynamics, print fidelity, and shape retention through complex mechanisms that govern material flow behavior during the printing process and subsequent structural stability [34,35]. For instance, extrusion performance in piston-driven bioprinting hinges on viscosity: excessive levels hinder flow and elevate cell-damaging pressure, while insufficient viscosity weakens structural integrity and shape fidelity Also, the shear-thinning behavior of bioink facilitates smooth extrusion by reducing apparent viscosity under the high shear conditions within printing nozzles, that typically range from 200 to 400 s^−1^ for 250 μm diameter orifices, but it maintains higher viscosity at rest to support printed structures. Furthermore, yield stress plays a critical role in determining both extrudability and shape maintenance, as materials must exceed this threshold to initiate flow during extrusion yet possess sufficient yield strength to resist gravitational deformation and support overhanging features in multi-layer constructs. In this way, the relationship between consistency index (K) and extrusion pressure follows predictable linear correlations, enabling rational prediction of printing parameters from rheological measurements and reducing the trial-and-error optimization traditionally required for new bioink formulations. A very important feature to take into account is related to the viscoelastic properties of the material as its further influence extrusion through their impact on elastic recovery and die swell effects, where materials with pronounced elastic character may exhibit dimensional instability immediately post-extrusion, requiring a careful balance of storage and loss moduli to achieve optimal flow and retention characteristics [36,37,38].

Therefore, it is clear that the print fidelity and shape retention in extrusion-based bioprinting are governed by the complex balance between yield stress, recovery kinetics, and gelation behavior that determines how effectively printed structures maintain their intended geometry throughout the fabrication process [39,40]. Thus, materials with inadequate yield stress undergo excessive spreading and fusion between adjacent deposited lines, which compromises printing resolution and eliminates the designed porosity critical for nutrient transport and cellular infiltration—both essential for the success of patient-specific tissue engineering constructs. Moreover, the timing of structural recovery is crucial during multi-layer printing, as thixotropic rebuilding must occur rapidly to support successive layers without triggering premature gelation that could hinder further material deposition. Quantitative analyses linking rheological parameters to print quality metrics indicate that optimal shape fidelity depends on yield stress values that are high enough to preserve the integrity of deposited lines, yet low enough to maintain sufficient flow for smooth extrusion. Similarly, temperature-dependent rheological behavior introduces additional complexity, as thermo-reversible bioink must retain suitable viscosity during heated extrusion while rapidly developing sufficient mechanical strength upon cooling to ambient printing bed temperatures. To address these challenges, rheological modifiers—such as Carbopol are commonly added to bioink to expand the printability window of otherwise unsuitable formulations, enabling desirable flow characteristics without compromising biological functionality. Furthermore, researchers are increasingly employing advanced modeling techniques, including computational fluid dynamics, to predict shear stress distributions within printing systems. These approaches facilitate the optimization of processing conditions to minimize cellular damage while maximizing print fidelity through strategic manipulation of rheological parameters [35,41,42,43].

It is important to note that rheological parameters impact bioprinting methods differently, owing to distinct mechanisms of material deposition and structure formation across technologies [44,45]. The main commercially available bioprinting techniques include: extrusion-based bioprinting, which employs pressure-driven flow to deposit continuous filaments of cell-laden hydrogels; inkjet bioprinting, which uses thermal or piezoelectric forces to eject bioink droplets onto a substrate; laser-assisted bioprinting, where pulsed laser energy transfers droplets from a donor ribbon to a receiving surface; and vat photopolymerization methods such as stereolithography (SLA) and digital light processing (DLP), which solidify layers of photo-sensitive bioink through patterned light exposure [46,47]. Each bioprinting technique places distinct demands on the physical properties of bioink, making rheological influence highly dependent on the chosen method. For example, extrusion-based bioprinting requires bioink with pronounced shear-thinning behavior, suitable yield stress, and rapid recovery to maintain shape fidelity and support multi-layered structures after deposition. In contrast, inkjet bioprinting demands low-viscosity bioink to ensure smooth droplet formation and prevent nozzle clogging, while laser-assisted systems also favor low-viscosity materials for precise droplet transfer and high cell viability. Vat photopolymerization techniques like SLA and DLP stand apart, as they necessitate ultra-low viscosity bioink to enable rapid and uniform recoating of the resin surface between each layer and to allow efficient light penetration for photopolymerization. In these light-based systems, the focus shifts from mechanical stability during deposition to the kinetics of photoinitiated gelation and the ability to achieve high-resolution features without flow-induced artifacts [47]. To date, the most striking contrast emerges when comparing extrusion-based and SLA bioprinting. Extrusion methods rely on rheological profiles that balance pressure-driven flow with rapid solid-like recovery, enabling the layer-by-layer construction of complex, self-supporting geometries. In contrast, SLA prioritizes Newtonian, low-viscosity flow to facilitate smooth resin movement and relies on rapid increases in elastic modulus upon light exposure to define structure—rather than mechanical stability during deposition. As a result, the ideal bioink for extrusion would be unsuitable for SLA, and vice versa, underscoring the necessity of tailoring rheological properties to the specific requirements of each bioprinting technology.

## 3. Biological Functionality of Bioinks

Bioinks play a crucial role in 3D bioprinting, serving as the medium that supports cell survival, growth, and functional development within printed constructs. To ensure successful tissue engineering applications, bioinks must meet several key biological and mechanical requirements.

### 3.1. Requirements for Cell Viability

Ensuring cell viability is a fundamental requirement in 3D bioprinting, as the success of bioprinted tissues depends on the ability of cells to survive the printing process and maintain their biological functions. Bioinks must be non-toxic and biocompatible, allowing cells to endure mechanical stress, extrusion forces, and post-printing culture conditions without compromising their integrity. Several factors influence cell viability, including biomaterial selection, printing parameters, sterilization methods, and degradation kinetics. The choice of biomaterials used in bioink formulation plays a crucial role in determining cell survival rates. Natural polymers such as collagen, alginate, gelatin, and hyaluronic acid are widely used due to their biocompatibility and ability to mimic the extracellular matrix (ECM) [48]. Handral et al. [49] reported that synthetic polymers like polyethylene glycol (PEG) and polycaprolactone (PCL) offer tunable mechanical properties but may require additional modifications to enhance biological compatibility. The chemical composition of bioinks must be carefully designed to avoid cytotoxic effects, ensuring that cells remain viable throughout the printing and maturation process. Maintaining sterility is essential to prevent contamination and ensure long-term cell survival. Previous study recommended that bioinks must undergo sterilization using methods such as UV irradiation, filtration, or autoclaving, depending on the sensitivity of the biomaterials [50]. However, excessive sterilization can alter the bioink’s properties, affecting cell adhesion and proliferation. Researchers are exploring gentle sterilization techniques that preserve bioink functionality while eliminating microbial contaminants. During bioprinting, cells experience shear stress and mechanical compression, which can impact their viability. Chen et al. [51] found that shear-thinning bioink, which reduce viscosity under shear stress, help minimize damage during extrusion. Additionally, optimizing printing speed, nozzle diameter, and extrusion pressure can significantly improve cell survival rates [52]. Studies have shown that lower extrusion pressures and controlled deposition rates enhance cell viability while maintaining print fidelity [53]. Bioinks must degrade at a rate that allows cells to deposit their own ECM while maintaining structural integrity [49]. If degradation occurs too quickly, the printed constructs may collapse before tissue maturation. Conversely, slow degradation can hinder cell migration and tissue remodeling. Researchers are developing bioinks with tunable degradation properties, incorporating enzymatic or hydrolytic degradation mechanisms to support long-term tissue viability.

### 3.2. Requirements for Proliferation

For effective tissue formation, bioinks must support cell proliferation, ensuring that encapsulated cells can divide, expand, and populate the printed scaffold efficiently. Several factors influence proliferation, including growth factor incorporation, bioinks porosity, and degradation kinetics. Bioinks must be enriched with growth factors and essential nutrients that stimulate cell cycle progression and enhance proliferation. Growth factors such as fibroblast growth factor (FGF), epidermal growth factor (EGF), and transforming growth factor-beta (TGF-β) play crucial roles in regulating cell division and differentiation. For instance, silk fibroin-alginate methacrylate-gelatin enriched bioinks promoted cell proliferation in 3D-bioprinted constructs. Additionally, bioinks can be supplemented with amino acids, glucose, and oxygen-releasing agents to sustain cellular metabolism and promote expansion [54]. For a good proliferation, the porosity of a bioinks is a key determinant of cell migration and spatial distribution within the scaffold. A well-designed bioinks should exhibit interconnected pores that allow cells to spread, interact, and form tissue-like structures [55]. Studies have shown that highly porous bioinks facilitate nutrient diffusion and waste removal, improving overall cell viability and proliferation [52,56,57]. Hydrogels such as alginate, gelatin, and silk fibroin have been widely used to create porous microenvironments that support cell growth [1,58,59]. In addition, bioinks must possess a controlled degradation rate, ensuring that as the scaffold degrades, new cells can replace it with their own ECM [51,60,61]. If degradation occurs too rapidly, the scaffold may lose its structural integrity before tissue maturation. Conversely, slow degradation can hinder cell migration and ECM deposition. Researchers are developing bioinks with enzymatic or hydrolytic degradation mechanisms, allowing for precise control over scaffold stability.

### 3.3. Requirements for Differentiation

Cell differentiation is a highly intricate biological process in which unspecialized cells transition into distinct cell types with specific functions. This transformation is guided by a combination of genetic programming and environmental signals. Differentiated cells are essential for the proper functioning of tissues and organs, ensuring they can perform specialized roles such as muscle contraction, nerve signaling, or oxygen transport. For bioinks—materials used in bioprinting to create tissue structures that successfully support and enhance cell differentiation, they must fulfill certain criteria.

#### 3.3.1. Cell Adhesion

Cells need to attach to the bioink matrix to maintain their position and interact with their surroundings, a prerequisite for proper differentiation [62,63,64,65]. Bioinks can be modified with cell adhesion ligands, such as RGD peptides, to enhance cell attachment and spreading [62]. The presence of these ligands promotes integrin binding, facilitating cell-matrix interactions crucial for cell differentiation. Other adhesion motifs, such as YIGSR and GFOGER, can also be incorporated to promote cell-specific adhesion [64]. Furthermore, the surface properties of the bioink can be modified to enhance cell adhesion, for example, through plasma treatment or coating with ECM proteins like fibronectin and laminin [65,66]. Strong cell adhesion ensures that cells remain in contact with differentiation cues and can respond appropriately.

#### 3.3.2. Nutrient and Oxygen Transport

The bioinks must allow for the diffusion of nutrients and oxygen to cells, critical for maintaining cell viability and supporting energy-intensive differentiation processes. Pore size and interconnectivity within the bioinks structure are critical factors influencing transport properties. Hydrogels with high water content and interconnected pores generally exhibit better nutrient and oxygen exchange [57,67]. Techniques such as incorporating sacrificial materials (microparticles or fibers) that are later removed to create interconnected pores can enhance nutrient transport [68]. Additionally, the use of microfluidic channels within the bioprinted construct can provide direct nutrient delivery [69]. Oxygen carriers, such as perfluorocarbons, can also be incorporated into the bioinks to improve oxygen availability to cells [70]. Adequate nutrient and oxygen supply are essential for cells to execute the complex molecular processes involved in differentiation.

#### 3.3.3. Biochemical Signaling

Bioinks can be functionalized with growth factors and other signaling molecules to direct cell behavior and specifically guide differentiation pathways. Controlled release of these factors can be achieved through various methods, such as encapsulation within microparticles or covalent attachment to the bioinks matrix [71,72]. This allows for precise control over cell differentiation and tissue development. Growth factors like vascular endothelial growth factor (VEGF) for osteogenic and angiogenic tissue formation [73] and transforming growth factor-β (TGF-β) for cartilaginous tissues [74] can be incorporated. The incorporation of specific biochemical signals is a direct method to instruct cells to differentiate along desired lineages.

#### 3.3.4. Appropriate Mechanical Properties

The stiffness and elasticity of the bioinks can significantly influence cell fate, particularly cell differentiation. Cells respond to mechanical cues from their environment, and the bioinks must provide the appropriate mechanical signals to guide differentiation. It is noted that more rigid matrices have been shown to promote the osteogenic differentiation of mesenchymal stem cells (MSCs), while softer matrices are more conducive to neurogenic differentiation [75]. Furthermore, the mechanical properties of the bioinks can be adjusted by altering the polymer concentration, crosslinking density, and incorporating reinforcing agents such as nanoparticles or microfibers [76]. Additionally, methods like dynamic crosslinking can be employed to develop bioinks with mechanical properties that change over time, simulating the dynamic variations found in the native tissue environment [77]. It is essential to align the mechanical characteristics of the bioinks with those of the target tissue to effectively guide cell differentiation.

#### 3.3.5. Presence of Differentiation Factors

Growth factors, cytokines, and various signaling molecules play a crucial role in steering cell differentiation. Bioinks must be formulated to include these factors and release them in a regulated manner. The specific factors needed will vary based on the target cell type and differentiation pathway. For example, bone morphogenetic protein-2 (BMP-2) is frequently utilized to promote osteogenic differentiation [78]. Other notable examples are nerve growth factor (NGF) for neuronal differentiation and hepatocyte growth factor (HGF) for liver tissue engineering [79]. The delivery of these factors can be enhanced by employing combinations of growth factors or by integrating small molecules that influence cell signaling pathways [80]. The addition of suitable differentiation factors serves as a direct and effective strategy for directing cell fate.

#### 3.3.6. Microenvironment

The three-dimensional structure of the bioinks offers a more physiologically relevant setting for cell differentiation when compared to conventional two-dimensional culture. This 3D configuration facilitates essential interactions between cells and the extracellular matrix, which are vital for tissue development. Bioprinting provides precise control over the bioink’s 3D architecture, allowing for the fabrication of intricate tissue structures that closely resemble the native tissue environment [81]. The addition of microchannels, perfusable vascular networks, and spatially defined niches can further improve the physiological relevance of the bioprinted construct [82]. Co-culturing various cell types within the 3D framework can also enhance cell-cell interactions and boost tissue functionality. The 3D microenvironment closely mimics in vivo conditions, fostering more natural and effective cell differentiation.

### 3.4. Requirements for Tissue Maturation

The fundamental requirement for bioinks used in tissue maturation is influenced by various factors related to their practical applications. Generally, bioink designed for tissue maturation in 3D bioprinting must satisfy a series of interconnected criteria to ensure effective cell survival, differentiation, and long-term structural integrity. Additionally, biocompatibility is crucial factor when comes to bioink formulation to avoid any occurrence of immune responses and facilitate proper integration with the native tissue. Similarly, biodegradability is an important key factor to deliver therapeutic biomolecules in target specific manner for proper tissue regeneration. Additionally, mechanical properties, including elasticity and stiffness, should closely mimic those of the target tissue, providing support to the printed structure during maturation and fostering cellular interactions [83,84,85,86].

In the same time, printability is crucial for ensuring precise deposition and maintaining shape fidelity, with shear-thinning behavior being essential for smooth extrusion during the printing process while preserving architectural integrity after printing. To sustain cell viability and enhance metabolic functions throughout the construct, bioinks must facilitate efficient transport of nutrients and waste. Furthermore, the crosslinking mechanism should be biocompatible and allow for controlled gelation to create stable, three-dimensional environments that are favorable for cell growth.

Most importantly, bioinks need to facilitate cell adhesion and signaling, which necessitates the inclusion of biochemical signals like RGD peptides or growth factors to promote cellular attachment, migration, and lineage-specific differentiation. The biofunctionalization process using peptides and bioactive molecules improves cellular interaction and accelerates effective tissue maturation. A significant breakthrough in recent years has been the creation of hybrid bioinks—combinations of natural and synthetic polymers designed to enhance biological activity while ensuring reproducibility and mechanical stability. These hybrid formulations provide better printability and flexibility, making them especially suitable for intricate tissue engineering projects. Ultimately, a successful bioink should have the ability to remodel the matrix, allowing the embedded cells to reorganize their surroundings and gradually substitute the scaffold with their own ECM. Ongoing research is crucial to further our understanding of how the composition of bioinks affects cellular behavior and tissue development, which is vital for the advancement of functional, clinically applicable constructs.

#### 3.4.1. Natural vs. Synthetic Polymers

##### Natural Polymers in Bioinks

The raw materials for fabrication of bioinks play a crucial role in their biological functions such as tissue maturation, cell viability, differentiation, and structural integrity (Table 3). In this sense, natural polymers are considered as exceptional raw materials for bioink formulation due to their inherent biocompatibility, biodegradability, and capacity to interact with cells. Most importantly, they often mimic the components of the ECM, providing cells with a microenvironment that is more physiologically relevant. However, their limited mechanical properties and printability can sometimes present challenges in practical applications and thus can be addressed through composite formulation approaches. Currently, several polymers such as alginate, collagen, gelatin, hyaluronic acid (HA), fibrin and chitosan are attracted considerably for bioink formulation. Alginate is a brown algae derived polysaccharide, which is widely used for bioink formulation due to its rapid gelation in the presence of divalent cations like Ca^2+^. Though it is an exceptional material for bioink, it lacks cell adhesion motifs, which need functional modification. Similarly, collagen, the most abundant human protein, provides excellent cell adhesion and remodeling capabilities, which makes it more suitable material for bioink formulation. However, its limited mechanical strength needs further enhancement for appropriate cell culture. Gelatin, a denatured collagen, maintains numerous advantageous characteristics of collagen owing to its hydrophilic properties, making it suitable for formulating bioinks. Additionally, the modification of gelatin with methacrylate (Gelatin methacrylate, GelMA) increases its applicability in intricate organ printing by providing improved mechanical stability via photo-crosslinking. Another natural polymer, HA, a glycosaminoglycan in ECM, is known for its water retention capabilities and its role in cell signaling. It is often modified to improve its mechanical properties and printability. As a natural scaffold formed during the blood clotting process, fibrin is an excellent polymer for cell adhesion and migration, thus widely used for bioink formulation. Lastly, chitosan, a deacetylated chitin, is another natural polymer widely used for bioink formulation due to its antibacterials, anti-inflammatory, wound healing and tissue regenerative properties.

##### Synthetic Polymers in Bioinks

On the other hand, synthetic polymers also play a crucial role in the formulation of bioinks for 3D bioprinting, offering mechanical stability, customizable degradation rates, and controlled bioactivity. Most importantly, unlike natural polymers, synthetic polymers provide improved reproducibility and adaptability, making them most suitable polymers for tissue engineering and regenerative medicine. A range of synthetic polymers is frequently employed in bioink formulations, each with unique advantages: Poly(ethylene glycol) (PEG) is a highly biocompatible and hydrophilic polymer, extensively used in hydrogel-based bioink for cell encapsulation. PEG can be modified with RGD peptides to boost cell adhesion. Poly(lactic-co-glycolic acid) (PLGA) is a biodegradable polymer with adjustable degradation rates, offering mechanical support for bone and cartilage tissue engineering. It is often blended with natural polymers to enhance bioactivity. Polycaprolactone (PCL) is a slowly degrading polymer that is perfect for long-term scaffolds. It is frequently used in hard tissue applications, such as bone and dental regeneration. Polyurethane (PU) is widely used in vascular tissue engineering due to its elasticity, durability and cell compatibility. Polydimethylsiloxane (PDMS) is a flexible and biocompatible polymer, widely used in soft tissue engineering due to its excellent oxygen permeability, making it ideal for cell cultures.

**Hybrid approaches in bioink formulation:** To overcome the above drawbacks, the combination of natural and synthetic polymers in bioink formulation can be a well-rounded method for tissue engineering, utilizing the biocompatibility of natural polymers alongside the mechanical stability provided by synthetic polymers. This hybrid approach improves cell adhesion, bioactivity, and structural integrity, making it particularly suitable for tissue maturation and regeneration. The major advantages of hybrid bioinks are enhanced biocompatibility (cell-adhesive motifs from natural polymers and controlled degradation and mechanical support from synthetic polymers), improved printability (rheological stability for accurate deposition during 3D bioprinting), optimized mechanical properties (stiffness of native tissue for cell differentiation and ECM deposition) and synergistic effects (improved bioactivity through integration of natural and synthetic components for tissue maturation) [87]. Furthermore, the incorporation of bioactive molecules, such as growth factors or cell-adhesive peptides, enhances cellular interactions and encourages tissue-specific remodeling. Recent research indicated that integrated decellularized ECM-synthetic polymers (PEG/PCL) hybrid systems enhanced vascularization, osteogenesis, and long-term tissue viability [87]. These findings highlighted the critical nature of rational hybrid bioink design, where material selection, crosslinking strategies, and cell compatibility are aligned to promote functional tissue maturation.

**Hybrid bioinks requirements for tissue maturation:** Hybrid bioinks developed for tissue maturation must meet several essential criteria of biological and physicochemical requirements to ensure the successful integration and functionality of bioprinted constructs. These bioinks need to demonstrate high biocompatibility, enabling embedded cells to survive, proliferate, and differentiate without provoking an adverse immune response. The degradation profiles of both natural and synthetic components must be synchronized to allow for gradual scaffold resorption while supporting ECM deposition. Mechanical properties such as elasticity and stiffness should be customized to replicate the native tissue microenvironment, thereby facilitating cell signaling and maintaining structural integrity during maturation. Moreover, hybrid bioinks must exhibit optimal rheological properties such as shear-thinning behavior and rapid gelation to guarantee printability and shape confirmative.

#### 3.4.2. Cell-Adhesive Motifs

Cell-matrix interactions play a crucial role in regulating cellular signals that lead to specific mechanisms, ultimately achieving the desired outcomes. Therefore, incorporating cell-adhesive motifs via surface functionalization on the surfaces of bioinks can effectively regulate cell-matrix interactions, thereby influencing the necessary regulatory signals. Generally, short sequences of amino acids such as RGD, YIGSR, IKVAV and GFOGER that resemble natural motifs found at binding sites in the ECM are recognized as effective cell-adhesive motifs. Consequently, the integration of these adhesive motifs into bioink has improved cell attachment, spreading, and the critical signaling pathways required for tissue maturation.

##### Key Cell-Adhesive Motifs and Their Functions

RGD (Arginine-Glycine-Aspartic acid) motif is well-known and widely recognized cell adhesive motifs, and empirical evidence demonstrate the possible interaction of RGD with several integrins (alpha and beta). These transmembrane receptors facilitate specific cell-ECM interactions. RGD promotes cell adhesion, spreading, and migration, subsequently influencing cell survival and differentiation. Different integrin subtypes display varying affinities for RGD, allowing for tailored cellular responses. Next to RGD, YIGSR (Tyrosine-Isoleucine-Glycine-Serine-Arginine, a crucial element of basement membranes) derived from laminin is a well-known cell-adhesive motif that supports cell adhesion and proliferation. Empirical evidence claimed that YIGSR influences cell differentiation and tissue organization, particularly in neural and epithelial tissues by interacting with laminin receptors. In addition, another cell-adhesive motif IKVAV (Isoleucine-Lysine-Valine-Alanine-Valine) from laminin, accelerates cell proliferation, cell adhesion, differentiation, and angiogenesis through binding to laminin receptors, especially in neural and endothelial cells. There is also well-studied cell-adhesive motif GFOGER (Glycine-Phenylalanine-Hydroxyproline-Glycine-Glutamate-Arginine) derived from collagen enhances cell adhesion and collagen fibrillogenesis through specifically binding to α1β1 and α2β1 integrins in bone and connective tissues.

##### Impact on Tissue Maturation

The types and concentration of cell-adhesive motifs in bioink significantly influence tissue maturation through various mechanisms. For instance, cell-adhesive motifs provide specific binding sites for cells and facilitate cell attachment to the bioink scaffold, thereby creating target-specific drug delivery. This attachment is crucial for cell spreading, essential for cell survival and functionality. Also, cell-adhesive motifs can promote cell proliferation by establishing a supportive microenvironment, leading to a higher cell density within the bioprinted structure. Certain motifs can guide cell differentiation towards specific lineages. For example, YIGSR and IKVAV can encourage neural differentiation, while GFOGER can enhance osteogenic differentiation. These motifs can stimulate cells to produce their own ECM, further supporting tissue maturation and integration. Overall, motifs can assist in the formation of organized tissue structures by promoting interactions between cells and the matrix.

#### 3.4.3. Considerations for Bioink Design

The major consideration for optimal bioink design depends on several factors such as motif selection, concentration, presentation and degradation rate. First and foremost, the choice of motifs must be carefully tailored to the specific cell type and target tissue, as different cells exhibit distinct integrin expression patterns and respond variably to biochemical cues. Also, it is very important to determine the ideal concentration of these motifs required for empirical validation, since insufficient levels may fail to elicit meaningful cellular responses, whereas excessive concentrations can lead to receptor saturation, diminishing functional engagement and potentially disrupting downstream signaling pathways. Equally important is the mode of motif presentation within the bioink matrix. Whether the motif is covalently conjugated to the polymer backbone or incorporated as a freely soluble peptide, its spatial accessibility and stability directly influence its bioactivity and interaction with cell surface receptors. Furthermore, the degradation rate of the bioink plays a pivotal role in regulating the temporal availability of these motifs. A well-calibrated degradation profile ensures that motifs remain accessible during critical phases of tissue development, while simultaneously allowing for scaffold resorption and replacement by native extracellular matrix components. Collectively, these parameters must be considered to create bioink that not only support initial cell adhesion and viability but also guide long-term tissue maturation and functional integration.

#### 3.4.4. Decellularized Extracellular Matrix (dECM)

dECM has emerged as a highly promising biomaterial in tissue engineering due to its intrinsic biocompatibility, biodegradability, and its ability to promote essential cellular behaviors such as adhesion, proliferation, and differentiation. When processed into printable bioink—typically through solubilization and gelation—dECM enables the fabrication of complex three-dimensional constructs that closely mimic the biochemical and structural characteristics of native tissue microenvironments. However, the successful application of dECM-derived bioink in achieving complete and functional tissue maturation requires meticulous consideration of several interdependent factors. First, the bioink composition must be optimized to preserve key ECM components such as collagen, glycosaminoglycans, and growth factors, which are vital for cellular signaling and matrix remodeling. Second, crosslinking strategies—whether physical, chemical, or enzymatic—must be carefully selected to balance mechanical stability with cytocompatibility, ensuring that the bioink maintains its shape without compromising cell viability or bioactivity. Lastly, the cell source plays a pivotal role; whether using primary cells, stem cells, or differentiated cell lines, the chosen cells must be compatible with the dECM scaffold and capable of responding to its biochemical cues to drive tissue-specific maturation. Together, these elements form the foundation for engineering biologically functional tissues using dECM-based bioink.

**Bioink composition:** The composition of decellularized extracellular matrix (dECM) bioink plays a pivotal role in guiding tissue maturation, as it directly influences the biochemical, mechanical, and cellular dynamics of the engineered construct. A critical first step involves selecting an appropriate ECM source, whether derived from specific organs, tissue types, or species, since each origin imparts a unique composition of structural proteins, GAGs, and growth factors. The decellularization technique employed must be carefully optimized to preserve these bioactive components while effectively removing cellular debris, thereby maintaining the functional integrity of the dECM bioink. It is also important that the concentration of dECM is within the optimum bioink formulation, which greatly impacts the outcomes. For instance, higher concentrations typically yield stiffer hydrogels, which can enhance mechanical stability but may impede cell spreading, migration, and differentiation. Therefore, fine-tuning the dECM concentration is essential to achieve a balance between structural support and cellular permissiveness, ultimately fostering robust cell-matrix interactions.

To further enhance tissue maturation, the incorporation of targeted additives and supplements is often necessary. Bioactive molecules such as transforming growth factor-beta (TGF-β) and bone morphogenetic protein-2 (BMP-2) can stimulate lineage-specific differentiation and promote endogenous ECM synthesis. Additionally, GAGs like hyaluronic acid contribute to bioink hydration and facilitate cell motility, creating a more favorable microenvironment for tissue development. The selection and dosage of these additives must be tailored to the specific tissue type and desired cellular outcomes, ensuring that the bioink supports both immediate cell viability and long-term functional integration.

**Crosslinking strategies:** Crosslinking is a fundamental process in the stabilization of dECM bioink, as it directly influences their mechanical integrity, printability, and biological performance during tissue construct development. Among the various strategies, physical crosslinking such as temperature-induced gelation offers a cytocompatible approach that preserves the native bioactivity of the dECM. This method avoids the use of chemical agents, thereby minimizing cytotoxicity and maintaining the biochemical cues essential for cell signaling. However, bioink stabilized solely through physical means often exhibit limited mechanical strength and structural stability, which may restrict their application in load-bearing or long-term tissue constructs. To address these limitations, chemical crosslinking techniques are frequently employed. Chemical crosslinkers such as glutaraldehyde and genipin can significantly enhance the mechanical robustness of dECM bioink by forming covalent bonds between matrix components. Despite their effectiveness, these chemical crosslinkers pose potential risks, including cytotoxicity and alteration of the dECM’s native bioactivity. Therefore, it is imperative to carefully optimize crosslinking conditions—such as concentration, exposure time, and reaction environment—to mitigate adverse effects while achieving the desired mechanical properties.

As a more biocompatible alternative, enzymatic crosslinking has gained attention for its ability to stabilize dECM bioink without compromising cellular viability. Enzymes like transglutaminase catalyze the formation of covalent bonds between glutamine and lysine residues within the ECM, resulting in hydrogels that are both structurally stable and biologically favorable. This approach supports the preservation of bioactive motifs and facilitates cell-matrix interactions, making it particularly suitable for applications in regenerative medicine and tissue maturation. Ultimately, the choice of crosslinking strategy must be guided by the specific requirements of the target tissue, balancing mechanical demands with biological compatibility to ensure successful construct formation and functional integration.

**Cell source:** The selection of an appropriate cell source is a critical determinant in achieving successful tissue maturation within bioprinted constructs. Primary cells, which are directly harvested from the target tissue, offer a distinct advantage of being phenotypically and functionally aligned with native cells, thereby enhancing the physiological relevance of the engineered tissue. However, their limited availability and restricted proliferative capacity pose significant challenges for large-scale applications and long-term culture. In contrast, stem cells, such as mesenchymal stem cells (MSCs) and induced pluripotent stem cells (iPSCs), provide a more versatile and scalable alternative. These cells possess the ability to self-renew and differentiate into multiple lineages, enabling the fabrication of complex, multi-cellular tissue constructs. Nonetheless, the differentiation process must be precisely regulated to ensure the generation of specific cell types required for functional tissue development, as uncontrolled differentiation may compromise construct fidelity and performance.

It is also important to consider the dynamic interplay between cells and the dECM bioink. Effective cell–ECM interactions are essential for guiding cellular behavior, including adhesion, migration, and response to biochemical cues embedded within the matrix. The ECM serves not only as a structural scaffold but also as a reservoir of signaling molecules that influence cell fate decisions. Optimizing these interactions through appropriate matrix composition, mechanical properties, and presentation of bioactive motifs is vital for promoting cell survival, proliferation, and lineage-specific differentiation, ultimately driving the maturation and functionality of the engineered tissue.

## 4. The Rheology Trade-Off in Bioink Design: Balancing Printability and Functionality

**Figure 2:**
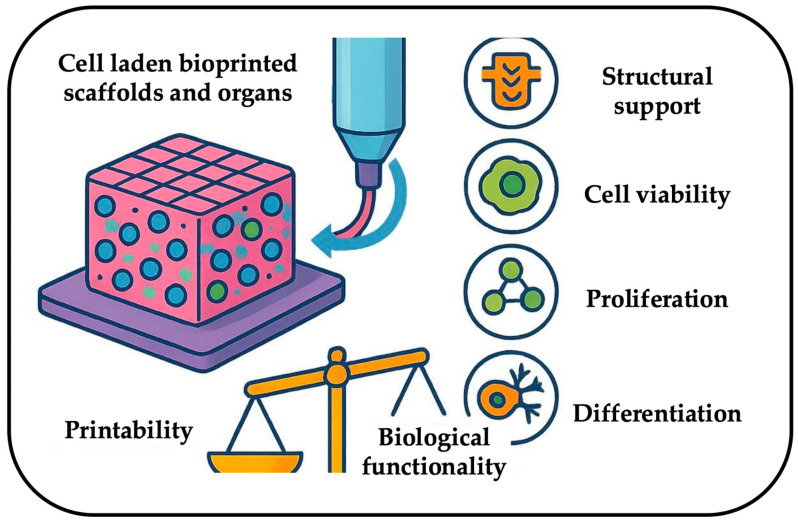
Schematization of the interlink between printability & biological functionality in cell-laden bioprinted scaffolds and organs.

A key challenge in the development of bioink for 3D bioprinting is achieving a careful balance between rheological and mechanical properties alongside biological performance (Figure 2). To attain optimal printability and shape fidelity, it is often necessary to customize the bioink composition and crosslinking methods; however, these modifications can negatively impact cell viability and functionality. For instance, Yue et al. [88] revealed that increasing photo-crosslinking in GelMA bioink improved mechanical stability but impeded cell migration and proliferation, highlighting the necessity of precisely adjusting crosslinking density to facilitate tissue development. Similarly, Webber and Shull [89] disclosed that the relationship between viscosity, print speed, and applied pressure influences print fidelity in alginate hydrogels, while Kafili et al. [90] demonstrated that higher levels of decellularized amniotic membrane (dAM) enhanced mechanical strength but diminished cell proliferation-again stressing the significance of compositional optimization.

Innovative strategies have been developed to tackle these trade-offs. For instance, Soliman et al. [91] presented a dual-step crosslinking system for gelatin-based bioink, which allows for precise control of viscosity and enhanced shape retention, leading to constructs that exhibit both high fidelity and excellent cell viability. In another study, the addition of nanomaterials, like graphene oxide, has further improved mechanical properties without sacrificing cytocompatibility [92]. However, Zandi et al. [93] disclosed that shear-thinning bioink, although advantageous for extrusion, subjected cells to mechanical stress could impact their viability. Das et al. [94] showed that bioink derived from decellularized ECM provide superior biological performance but necessitate further crosslinking to achieve mechanical stability. Paxton et al. [95] emphasized the need for standardized methods to evaluate printability and shape fidelity, underscoring the importance of rheological assessment and predictive modeling in the design of next-generation bioink.

Composite and hybrid bioink incorporating nanomaterials or granular microgels have demonstrated potential in addressing conventional limitations listed above. For instance, Rasouli et al. [96] examined nanofillers using carbon nanotubes, graphene oxide, and gold nanoparticles for improved conductivity, strength, and cell–matrix interactions, while facilitating stimuli-responsive behavior. Granular hydrogel bioink, as fabricated by Tuftee et al. [97] provided inherent porosity and dynamic crosslinking, which supported nutrient transport and immunomodulation. These materials possessed shear-thinning and self-healing attributes, allowing for high-fidelity extrusion and maintaining robust cell viability.

Recently, supportive strategies including sacrificial and support materials, have broadened the capabilities of bioprinting. Engineered support baths and sacrificial inks enable the printing of soft bioink into intricate geometries. Brunel et al. [98] reported that these materials offered temporary structural support and eliminated after printing without affecting cell viability. In this sense, gelatin, Pluronic F127, and Carbopol substances improved porosity and vascularization, which are essential for tissue maturation, as highlighted by Yeh et al. [99].

At the cutting edge of bioink development, dynamic and stimuli-responsive ability play a crucial role in 4D bioprinting. These bioinks react to environmental signals—such as temperature, pH, light, or magnetic fields—allowing for precise control over cell behavior in both space and time. Neumann et al. [100] demonstrated that intelligent hydrogels can replicate the dynamic ECM, aiding in the creation of shape-morphing tissue constructs. Khalid et al. [101] highlighted the promise of shape-memory polymers and multi-material constructs for customized implants tailored to individual patients. In most recent times, the combination of artificial intelligence (AI) and machine learning (ML) is transforming the development of bioinks [102,103]. Oh et al. [35] presented a rheology-informed ML model that forecasts printing resolution by considering bioink composition and process parameters. Meanwhile, Lee et al. [104] showed that ML-driven design strategies could pinpoint optimal formulations by examining the connections between mechanical properties, printability, and cell viability. These innovations facilitate real-time process management, defect identification, and customized tissue fabrication—signifying a significant advancement towards automated, high-throughput bioprinting. All these above-mentioned strategies emphasize that there is no universal solution in bioink formulation; rather, successful strategies rely on a nuanced combination of material selection, crosslinking methods, and compositional tuning to create constructs that are both structurally robust and supportive of tissue development.

## 5. Case Studies and Applications

To demonstrate the practical relevance of key concepts, several major studies are presented as proof of concept.

### 5.1. Concept 1: Bioinks for Cartilage Regeneration

**Figure 3:**
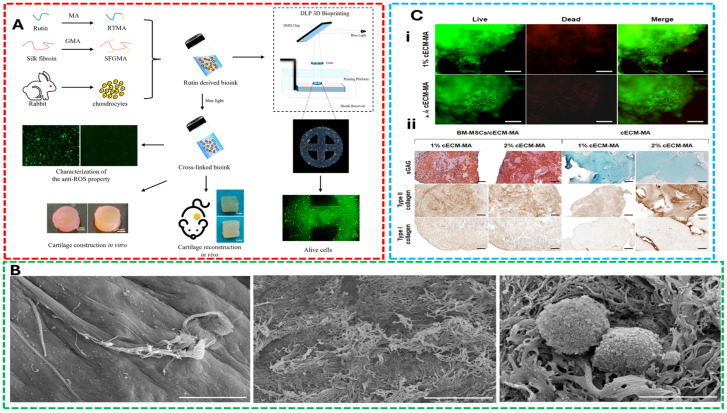
(**A**) Anti-oxidative bioinks (methacrylate-modified rutin-glycidyl methacrylate silk fibroin) for cartilage tissue engineering application [86]. (**B**) Visualization of cartilage with C28/I2 chondrocytes on decellularized scaffold by scanning electron microscopy. Cells attached to scaffold after 1 week in culture. (Scale bar = 20 µm) [105]. (**C**) Biocompatibility (**i**) Cell viability by Live/dead cell staining,(scale bars = 200 µm)) and (**ii**) chondrogenic inductivity of methacrylated cartilage extracellular matrix constructs stained for type I and II collagens and sGAGs (scale bars = 100 µm) [106]. Adapted under open access Creative Commons CCBY 4.0 license/ through open access permission from Refs. [86,105,106].

Gorroñogoitia et al. [85] fabricated alginate-based bioink by 3D Bioprinting for articular cartilage tissue engineering and evaluated the rheological and mechanical properties of alginate hydrogels, focusing on viscosity, cross-linking conditions, and structural integrity. The findings highlighted that molecular weight and M/G ratio significantly influence printability and scaffold resolution, emphasizing the need for precise control of bioink properties to achieve high-resolution tissue constructs. Furthermore, the study demonstrated that optimizing viscosity and shear-thinning behavior directly improves printability and filament resolution while maintaining chondrocyte viability. Their analysis emphasized the need for stable flow characteristics to support structural fidelity under extrusion conditions. Figure 3 shows anti-oxidative bioink composed of methacrylate-modified rutin-glycidyl methacrylate silk fibroin developed for cartilage tissue engineering applications, visualization of decellularized cartilage scaffolds seeded with C28/I2 chondrocytes, showing firm cell attachment and integration after 1 week of culture and evaluation of scaffold biocompatibility and chondrogenic potential.

Recently, Tong et al. [107] demonstrated that thermal and ionic modulation techniques effectively fine-tuned the viscoelastic properties of bioinks, enhancing extrusion consistency while promoting chondrogenic activity. Their study investigated the mechanical behavior, cell viability, and gene expression profiles of bioinks composed of sodium alginate–collagen type I and agarose. The results showed that these formulations significantly improved cell adhesion, proliferation, and cartilage-specific gene expression, owing to their favorable mechanical strength and biological functionality—making them promising candidates for cartilage tissue regeneration. Importantly, they correlated bioink gelation and crosslinking parameters to enhanced cell proliferation and scaffold integration, linking material chemistry to both rheological function and biological outcomes. O’Donnell et al. [108] optimized the printing parameters of hyaluronic acid-sodium alginate-collagen-chondroitin sulfate bioink for cartilage regeneration and disclosed that optimal printing 22G nozzle, highlighting how thermal and ionic modulation techniques can fine-tune viscoelastic properties for extrusion consistency while promoting chondrogenic activity.

These studies reveal how rheological properties such as viscosity, yield stress, and crosslinking kinetics are engineered not in isolation but alongside biological goals—namely chondrocyte survival, matrix deposition, and mechanical integration with host tissue. Importantly, the matrix–cell interaction is guided by not only biochemical cues but also by scaffold architecture dictated during the printing phase. For cartilage regeneration, the success of bioink design hinges on understanding these trade-offs: creating systems that are soft enough to protect cells from shear damage yet stiff enough to support anatomical structure and resist compressive loading. These applications underscore the need for integrative design strategies that align with the broader framework of printability-biocompatibility trade-offs of bioink.

### 5.2. Concept 2: Bioinks for Vascularized Tissue

**Figure 4:**
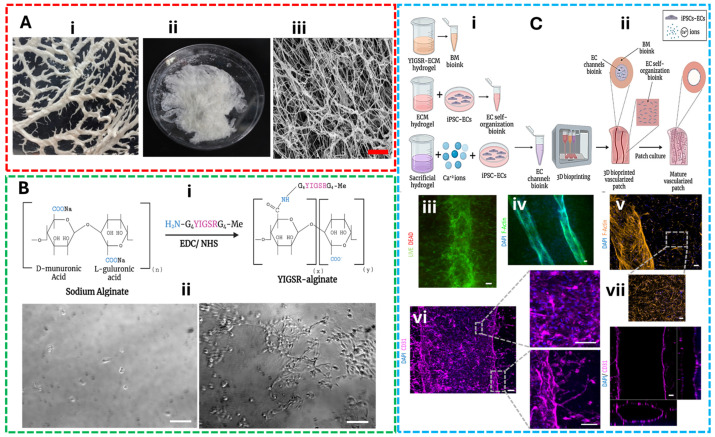
(**A**) Omentum decellularization and ECM hydrogel generation. (**i**)—Native omentum, (**ii**)—De cellularized omentum, (**iii**)—Representative hrSEM image of the hydrogel. Scale bar = 1 µm. (**B**) (**i**)—YIGSR-alginate gel generation schematics and (**ii**)—Adhesion of iPSCs-derived ECs to pristine (**left**) and modified alginate (**right**) sheets, 4 h post-seeding. (scale bar = 100 μm) (**C**) The 3D printed vascularized patches. (**i**) The bioinks that were used in the printing process. (**ii**) Schematics of the patch bioprinting process. (**iii**) Viability after printing. (**iv**) Representative fluorescence image of two printed large blood vessels stained for F-actin and DAPI after 14 days in culture (scale bar = 100 μm). (**v**) Representative fluorescence image of a printed large blood vessel and surrounding capillary-like structures stained for F-actin and DAPI after 14 days in culture (scale bar = 100 μm). (**vi**) Representative 3D confocal images of a 3D printed blood vessel immunostained for the endothelial cell marker CD31 after 14 days in culture, EC monolayer zoom-in image (**top**), a zoom-in image showing EC sprouting from the blood vessel wall (**bottom**) (scale bars = 100 μm). (**vii**) Representative 3D confocal images of a 3D printed blood vessel immunostained for CD31 after 14 days in culture (scale bar = 100 μm) [64]. Adapted under open access Creative Commons CCBY 4.0 license/through open access permission from Ref. [64].

Recent studies on vascularized tissue engineering underscore the importance of tailoring bioink properties not only for mechanical support but also for promoting perfusable networks and functional integration—core principles that align with the trade-offs discussed throughout this review. Machour et al. [109] developed a stiff, hybrid vascularized bone tissue by reinforcing bioink composed of thermosensitive poly(lactic-co-glycolic) acid and hydroxyapatite microparticles with a cell-laden ECM-based hydrogel and reported high cell viability, vascular network formation, and osteogenic differentiation in rat femoral defect models compared to acellular controls. This study highlighted that increased elastic modulus supports structural fidelity under load while still permitting vascular patterning through dual-material bioprinting. This reveals a strategic rheological compromise—engineering stiffness for shape retention while modulating flow properties for endothelial cell viability and vascular perfusion. Salg et al. [110] reviewed pre-, intra-, and post-printing strategies of bioink formulations including bioink composition, bioprinting platforms, and material deposition techniques to enhance vascularization in bioartificial tissues and emphasized bioconvergence approaches, such as computer simulation and AI-driven vascular tree modeling, to optimize vascularized parenchymal tissue fabrication. Their findings show that porosity and nutrient diffusion are tightly linked to rheological behavior and bioink crosslinking kinetics, influencing both printability and neovascularization success. Recently, researchers have developed stem cell-encapsulated, photocrosslinkable, shear-thinning alginate microgel (SSAM) bioink for vascularized bone tissue regeneration. SSAM was bioprinted into complex 3D structures, serving as a supporting bath for prevascular network formation. Their study relies on the synergistic balance between bioink microporosity, low extrusion stress, and high cellular density and concluded that these pre-vascularized constructs exhibited mechanical stability and long-term osteogenic differentiation in mouse calvarial bone defect models, demonstrating successful vascular integration [111]. Together, these examples illustrate that vascularization is not a downstream biological process but an upstream material design challenge. They reinforce the need to integrate rheological tuning, scaffold architecture, and cellular compatibility in bioink formulation—especially for complex tissues like bone and parenchyma. These evidences supported that the effective bioink strategies for vascularized tissues must reconcile mechanical constraints with biological imperatives at the material level. To illustrate the multifaceted approach used in the design and evaluation of engineered vascular constructs, Figure 4 illustrates the complete workflow and outcomes of vascularized tissue engineering using decellularized omentum-derived ECM hydrogels and modified alginate matrices. It includes the fabrication process, enhanced adhesion of iPSC-derived endothelial cells, and successful formation of large and capillary-like blood vessels, supported by fluorescence and confocal imaging of printed constructs after 14 days in culture.

### 5.3. Concept 3: Bioinks for Bone Regeneration

Bioinks development for bone regeneration offers a compelling illustration of the trade-offs between rheological performance, printability, and biological functionality—the central themes of this review. Bone tissue engineering demands bioink that not only support osteogenic differentiation but also withstand mechanical loads and maintain structural fidelity during and after printing. Celikkin et al. [112] assessed the biological properties (optimal tissue integration) of 3D printed 5% GelMA in a rat condyle defect model for bone regeneration applications. This study demonstrated that optimized GelMA bioink supported mesenchymal stem cell (MSC) encapsulation and in vivo testing model showed optimal tissue integration without fibrotic encapsulation or inhibited bone formation, demonstrating that its tunable crosslinking and viscoelastic properties enabled high-resolution extrusion printing while supporting both in vitro and in vivo osteogenesis. The study emphasized how adjusting polymer concentration and photoinitiator levels directly influenced printability and scaffold stiffness—key rheological parameters that also affected cell viability and mineralization. Similarly, Badhe et al. [113] emerging bioink technologies for bone and cartilage, highlighting the integration of bioactive ceramics (hydroxyapatite, calcium phosphate) into polymer matrices to enhance osteoconductivity. These composite bioink often require careful balancing of viscosity and particle dispersion to avoid nozzle clogging while maintaining mechanical integrity and biological responsiveness. The study concluded that inject printer-based and extrusion-based 3D bio-printing lack of resolution and precise printing due to low droplet directionality and printing head aperture, which are overcome by more advanced acoustic-based, laser-based printers, and SLA techniques. In addition, several polymers were widely used in bioink fabrication for bone regeneration such as agarose, collagen, alginate, gelatin methacrylate, cellulose nanofibrils, methycellulose, polyvinyl alcohol, hydroxyapatite, hyaluronic acid, silk fibroin, and carboxymethyl chitosan [113].

**Figure 5:**
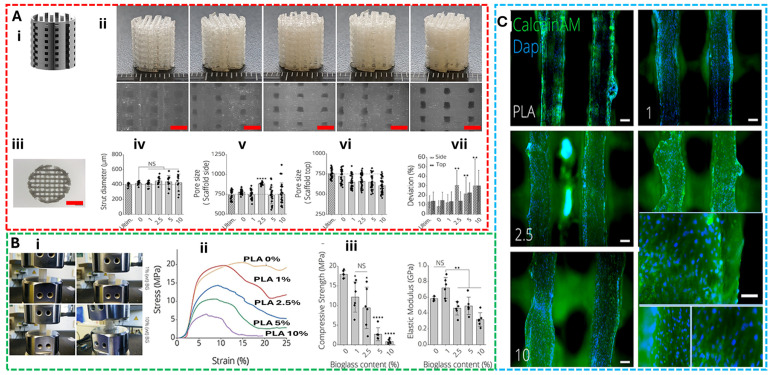
(**A**) 3D printed PLA-45S5 BG scaffolds. (**i**)—CAD render of PLA-BG scaffolds with a pore size of width = 750 μm, (**ii**)—FDM 3D printed PLA-BG scaffolds (PLA-0%, PLA-1%, PLA-2.5%, PLA-5%, and PLA-10%), Scale bars: 500 μm, (**iii**)—Top view light microscopy image of a PLA-BG scaffold. Scale bar: 2 mm. (**iv**–**vii**): Printability assessment and porosity analysis of PLA-BG scaffolds. ** *p* < 0.01, **** *p* < 0.0001 indicate statistical significant difference of means in comparison to 3D printed 0% BG PLA by one-way ANOVA or Welch’s *t*-test in pairwise comparisons of scaffold side pore diameter. (**B**) Mechanical properties of 3D printed PLA-45S5 BG scaffolds, (**i**)—Macroscopic images illustrating different failure modes of 1 and 10% (wt), (**ii**)—Qualitative stress strain diagram, (**iii**)—Compression strength and modulus of elasticity of 3D printed PLA-BG scaffolds. (**C**) Fluorescence microscopy images of Calcein AM (green)/DAPI (blue) stained MC3T3E1 on 3D printed PLA-BG scaffolds (Scale bar: 200 μm). Adapted under open access Creative Commons CCBY 4.0 license/through open access permission from Ref. [116].

Additional studies reinforce this synthesis. For example, Kumar et al. [114] explored nanocomposite bioink combining gelatin, polycaprolactone, and hydroxyapatite to improve both mechanical strength and osteoinductive potential. Li et al. [115] reviewed hydrogel-based bioink for bone tissue regeneration, emphasizing the importance of shear-thinning behavior and rapid gelation kinetics to support multilayered constructs with embedded stem cells. These works collectively demonstrate that successful bone bioink must reconcile the need for extrusion-friendly rheology with biomimetic matrix-cell interactions, enabling scaffold maturation and mineral deposition. By linking these applications to the broader framework of bioink design trade-offs, this section moves beyond summary to offer analytical insight, bone regeneration bioink exemplify how mechanical demands, biological cues, and printing constraints must be co-optimized to achieve functional tissue outcomes. To summarise, Figure 5 highlights the structural, mechanical, and biological performance of 3D printed PLA-45S5 bioactive glass composite scaffolds. It includes their design and printability, mechanical response under different compositions, and cellular compatibility, as evidenced by live cell staining on scaffold surfaces.

### 5.4. Concept 4: Decellularized Extracellular Matrix (dECM)–Bioinks

dECM–based bioinks offer a compelling solution to the challenge of balancing biological functionality with rheological and structural demands in 3D bioprinting. Unlike conventional hydrogel systems, dECM bioink retain native biochemical cues and microarchitectural features that promote cell–matrix interactions, making them highly suitable for tissue-specific regeneration. Wu et al. [117] fabricated cell-laden bioink containing dCECM, infrapatellar fat pad adipose-derived stem cells (IPFP-ADSC), gelMA and sodium alginate for chondrogenesis and found that bioink facilitated chondrogenic differentiation by providing a favorable cell matrix for stem cell proliferation and increase growth factors, while maintaining sufficient viscosity for extrusion-based printing. Their study highlights how tissue-derived bioink can be engineered to meet both biological and mechanical requirements for articular cartilage repair. Similarly, Xu et al. [118] investigated the effect of injectable photo-crosslinking cartilage decellularized extracellular matrix through 8 weeks of subcutaneous implantation in nude mice for cartilage tissue regeneration. The study observed dECM replicated characteristics of the cell’s native microenvironment and effective for filling and repairing cartilage defects by increasing collagen and GAG contents, showing that light-mediated gelation can enhance print fidelity without compromising the bioactivity of the matrix. This approach underscores the importance of crosslinking kinetics in tuning rheological behavior for precise deposition.

Behan et al. [106] developed a favorable rheological characterization of a methacrylated cartilage ECM-based bioink for cartilage tissue engineering with different percentages of porcine articular cartilage dECM and gelatin, both with and without methacrylation. Their findings suggested that higher concentrations of dECM might postpone the complete crosslinking of available sites within the bioink, leading to significantly influence the behavior of the bioink and concluding that the concentration of dECM in formulations was measurable impacts on rheological properties. Collectively, these studies reinforce the central thesis of this review that successful bioink design requires a nuanced understanding of how rheological properties, printability, and matrix–cell interactions intersect. dECM-based bioink exemplify this integration, offering biologically rich platforms that can be tailored for extrusion-based bioprinting while preserving the regenerative potential of native tissues.

## 6. Challenges and Future Perspectives

The advancement of 3D bioprinting from laboratory research to clinical reality faces significant drawbacks that require coordinated solutions across standardization, technological innovation, and regulatory frameworks. These challenges represent critical opportunities for establishing bioprinting as a transformative healthcare technology.

### 6.1. Standardization of Rheological and Biological Testing

The lack of standardized testing protocols represents one of the most pressing barriers to bioprinting advancement. Currently, rheological characterization of bioink varies dramatically between laboratories, with inconsistent reporting of fundamental parameters such as viscosity, shear-thinning behavior, and recovery kinetics. This variability hinders reproducibility and makes it difficult to compare research findings across institutions. Recent efforts by ASTM International have begun addressing this gap through the publication of “Standard guide for bioink used in bioprinting” (F3659), a document developed over six years by 35 leading experts from bioink manufacturers, regulatory bodies including the FDA, and research institutions like NIST [119]. This standard specifically focuses on extrusion bioprinting and establishes essential practices for bioink preparation, characterization, and post-printing evaluation. However, biological testing standardization faces equally complex challenges, particularly in establishing consistent protocols for cell viability assessment, sterility testing, and long-term functionality evaluation. Thus, the development of standardized quality control measures, including the verification of viscosity, pH, and cytotoxicity represents a crucial step toward ensuring reproducible biological outcomes. Furthermore, the incorporation of artificial intelligence and machine learning approaches, such as rheology-informed hierarchical machine learning models, offers promising solutions for predicting printing resolution and optimizing bioink formulations in a standardized manner.

Another huge challenge in bioink development lies in vascularization, scalability, and clinical translation. Indeed, vascularization remains the most significant technological barrier preventing the clinical translation of thick, functional tissues for real patient needs. To date, current bioprinting technologies struggle to create the complex vascular networks necessary for tissue survival beyond the 100–300 μm diffusion limit. However recent breakthroughs, including bioprinting combined with sacrificial gelatin microparticles, have demonstrated the ability to sustain cell viability nearly five times deeper than traditional methods by improving nutrient diffusion and enabling scalable perfusion strategies [120]. These advances incorporate computational modeling to predict oxygen distribution and inform optimal vessel spacing, which are essential capabilities for future clinical-scale tissue design.

For its part, the scalability challenges extend beyond vascularization to include manufacturing reproducibility and cost-effectiveness. Therefore, the real transition from laboratory-scale to industrial-scale production introduces complexities in maintaining consistent parameters such as temperature, pH, and nutrient supply, whereas managing increased shear stress can damage cells in large bioreactors. At present, automated bioprinting solutions are beginning to address these challenges by providing increased throughput, precision, and reproducibility compared to manual workflows. However, significant work remains in developing fully automated, high-throughput manufacturing processes that can produce clinically relevant tissue volumes, while maintaining quality and reducing costs [121].

### 6.2. Regulatory and Manufacturing Considerations

The regulatory landscape for bioprinting presents a complex, evolving framework that varies significantly across jurisdictions. In the United States, the FDA regulates bioprinted products through multiple pathways depending on their classification as medical devices (Class I–III requiring different levels of approval), biologics requiring Biologics License Applications, or combination products. The European Union employs a similarly complex approach through Advanced Therapy Medicinal Products (ATMPs) regulations, with oversight distributed across multiple agencies including the European Medicines Agency. A critical challenge lies in the interdisciplinary nature of bioprinting, which involves multiple stakeholders including 3D model designers, medical professionals, engineers, biologists, and regulatory experts. Currently, no comprehensive regulatory regime governs the entire bioprinting process, resulting in fragmented oversight that may not adequately address the unique risks and opportunities presented by this technology. The regulatory framework must balance innovation support with patient safety, requiring adaptive approaches that can evolve with technological advancement while maintaining rigorous safety standards [122].

It is worth mentioning that manufacturing considerations include both technical and ethical dimensions, including questions of product ownership, commercialization ethics, and accessibility. The high costs associated with bioprinting technology raise concerns about equitable access to these life-saving treatments, potentially creating disparities between different socioeconomic groups. Additionally, quality control and supply chain management present ongoing challenges, requiring robust systems to ensure consistency of raw materials and final products, while meeting stringent regulatory requirements [122].

### 6.3. Future Perspectives

Looking forward, the convergence of advanced biomaterials, automated manufacturing, predictive modeling, and adaptive regulatory frameworks will be essential for realizing bioprinting’s clinical potential. Key research areas identified as future hotspots include the development of new bioink with enhanced biological functionality, modification of extrusion-based bioprinting for improved cell viability and vascularization, application in organoids and in vitro models, and advancement of personalized regenerative medicine applications. Success in these areas will require tight collaboration among researchers, engineers, regulatory bodies, and industry stakeholders to overcome the multiple challenges that currently limit bioprinting’s clinical translation.

## 7. Conclusions

To conclude, it is clear that bioink design remains a pivotal yet complex challenge in 3D bioprinting, where the interplay between rheological performance and biological functionality dictates the success of printed constructs. As this review highlights, the rheology-biology trade-off manifests uniquely across bioprinting techniques, each imposing distinct demands on bioink properties. For extrusion-based methods, shear-thinning behavior and post-printing structural stability are critical, yet these requirements often conflict with the need for nutrient diffusion and cell migration. In contrast, light-based techniques prioritize photoreactivity and rapid crosslinking, which can inadvertently limit long-term cytocompatibility or dynamic matrix remodeling. Similarly, inkjet bioprinting favors low-viscosity bioink for droplet precision, but such formulations may lack the mechanical robustness needed for load-bearing tissues. As demonstrated widely in literature, recent advances in hybrid bioink, dynamic crosslinking strategies, and multi-material systems offer promising pathways to mitigate these trade-offs. For instance, interpenetrating network hydrogels and shear-thinning composites demonstrate how tailored material chemistry can decouple printability from biological constraints. However, the field still grapples with universal challenges standardization of bioink characterization, scalability for clinical translation, and the biomimetic integration of vascularization cues. In the near future, it is clear that moving forward, the development of “smart” bioink, responsive to mechanical, chemical, or biological stimuli, may connect these gaps by enabling spatially and temporally controlled microenvironments. Finally, as far as the recent advances demonstrated, no single bioink formulation or bioprinting technique will suffice for all applications. Indeed, success hinges on a technique-specific, application-driven approach that prioritizes the unique demands of target tissues, whether mechanically dynamic or metabolically active. It is very likely that by taking advantage of modern tools such as computational modeling, high-throughput screening, and novel biomaterial platforms, the next generation of bioink may finally reconcile printability with the nuanced biological requirements of functional tissue engineering, accelerating the transition from laboratory innovation to clinical reality.

## Figures and Tables

**Table 1:** Rheological parameters, ideal ranges of bioinks and their potential impact on its formulations.

Parameter	Definition	Printability Impact	Cell Viability Impact
Shear-thinning	Viscosity decreases under shear	Enables extrusion	Reduces shear stress
Yield stress (τ_0_)	Minimum stress to initiate flow	Prevents nozzle clogging	Limits mixing forces
G′/G″ ratio	Elastic vs. viscous dominance	Structural integrity	Mitigates post-print deformation
Thixotropy	Time-dependent viscosity recovery	Prevents layer sagging	Protects cells post-extrusion

**Table 2:** Key Rheological properties governing bioink performance.

Shear-Thinning Behavior	Yield Stress and Structural Fidelity	Viscoelasticity (G′, G″) Dynamics	Thixotropic Recovery
High viscosity at rest (prevents cell settling).	Below τ_0_: Elastic solid behavior (maintains shape post-printing).	Ideal bioink: G′ > G″ at rest (elastic dominance for shape retention).	Rapid structural rebuild (<30 s) prevents layer fusion issues.
Sharp viscosity drop under shear (enables extrusion through nozzles)	Above τ_0_: Viscous flow (enables extrusion).	G″ > G′ during extrusion (viscous flow).	Enhanced cell viability due to reduced sustained shear stress
Critical shear rate for optimal extrusion marked (avoids cell damage).	τ_0_ range (2–10 Pa) for cell-friendly mixing and filament integrity	Loss tangent (tan δ = G″/G′) range: 0.25–0.45 for balance	

**Table 3:** Natural vs. synthetic polymers in Tissue maturation.

Polymers	Natural	Synthetic
Advantages	Biocompatibility: Natural polymers are generally well-tolerated by the body, minimizing the risk of adverse immune responses. Bioactivity: Many natural polymers contain cell-binding motifs that promote cell adhesion, proliferation, and differentiation. Examples include RGD sequences in collagen and fibronectin. Biodegradability: Natural polymers are typically biodegradable, breaking down into non-toxic products that can be readily cleared by the body. Cell-Material Interactions: Natural polymers often mimic the natural ECM, providing a more physiologically relevant environment for cells.	Tunable Properties: Synthetic polymers can be precisely engineered to control their mechanical properties, degradation rate, and other characteristics. Reproducibility: Synthetic polymers are manufactured under controlled conditions, ensuring consistent batch-to-batch quality. Mechanical Strength: Synthetic polymers can be designed to provide high mechanical strength and durability. Processability: Synthetic polymers can be readily processed into a variety of shapes and structures using techniques such as electrospinning, 3D printing, and molding.
Limitations	Batch-to-Batch Variability: Natural polymers can exhibit significant variability in their composition and properties depending on the source and processing methods. Mechanical Properties: Natural polymers often lack the mechanical strength and durability required for load-bearing applications. Immunogenicity: Some natural polymers can elicit an immune response, particularly if they are derived from animal sources. Degradation Rate: Controlling the degradation rate of natural polymers can be challenging, potentially leading to premature scaffold collapse or delayed tissue regeneration. Processing Challenges: Some natural polymers can be difficult to process into desired shapes and structures.	Lack of Bioactivity: Synthetic polymers typically lack the inherent bioactivity of natural polymers, requiring modification to promote cell adhesion and differentiation. Biocompatibility Concerns: Some synthetic polymers can elicit adverse immune responses or release toxic degradation products. Hydrophobicity: Many synthetic polymers are hydrophobic, which can hinder cell adhesion and nutrient transport. Degradation Products: The degradation products of some synthetic polymers can be acidic, potentially causing inflammation and tissue damage.
Requirements for Tissue Maturation	Controlled Degradation: The degradation rate should match the rate of new tissue formation to provide structural support during the maturation process. Cell Adhesion Motifs: Incorporation of cell adhesion motifs (RGD) to promote cell attachment and spreading. Biocompatibility: Minimal inflammatory response and toxicity. Porosity: Sufficient porosity to allow for cell infiltration, nutrient transport, and waste removal. Mechanical Stability: Adequate mechanical strength to withstand physiological loads during the initial stages of tissue formation.	Biocompatibility: Non-toxic and non-immunogenic. Controlled Degradation: The degradation rate should be tailored to the rate of tissue formation. Mechanical Properties: Mechanical strength and elasticity should match the target tissue. Surface Modification: Surface modification with cell adhesion molecules (RGD peptides) to enhance cell attachment and spreading. Porosity: Interconnected pores to facilitate cell infiltration, nutrient transport, and waste removal. Sterilizability: Ability to be sterilized without compromising material properties.

## Data Availability

Not applicable.
